# Inducing respiratory complex I impairment elicits an increase in PGC1α in ovarian cancer

**DOI:** 10.1038/s41598-022-11620-y

**Published:** 2022-05-16

**Authors:** Monica De Luise, Manuela Sollazzo, Eleonora Lama, Camelia Alexandra Coadă, Licia Bressi, Maria Iorio, Beatrice Cavina, Luigi D’Angelo, Sara Milioni, Lorena Marchio, Stefano Miglietta, Sara Coluccelli, Greta Tedesco, Anna Ghelli, Silvia Lemma, Anna Myriam Perrone, Ivana Kurelac, Luisa Iommarini, Anna Maria Porcelli, Giuseppe Gasparre

**Affiliations:** 1 Department of Medical and Surgical Sciences (DIMEC), University of Bologna, 40138 Bologna, Italy; 2 Center for Applied Biomedical Research, University of Bologna, 40138 Bologna, Italy; 3 Centro Studi E Ricerca Sulle Neoplasie Ginecologiche (CSR), University of Bologna, 40138 Bologna, Italy; 4 Department of Pharmacy and Biotechnology (FABIT), University of Bologna, 40126 Bologna, Italy; 5 Division of Oncologic Gynecology, IRCCS Azienda Ospedaliero-Universitaria di Bologna, 40138 Bologna, Italy; 6 Interdepartmental Center of Industrial Research (CIRI) Life Science and Health Technologies, University of Bologna, 40064 Ozzano dell′Emilia, Italy

**Keywords:** Biochemistry, Cancer, Genetics

## Abstract

Anticancer strategies aimed at inhibiting Complex I of the mitochondrial respiratory chain are increasingly being attempted in solid tumors, as functional oxidative phosphorylation is vital for cancer cells. Using ovarian cancer as a model, we show that a compensatory response to an energy crisis induced by Complex I genetic ablation or pharmacological inhibition is an increase in the mitochondrial biogenesis master regulator PGC1α, a pleiotropic coactivator of transcription regulating diverse biological processes within the cell. We associate this compensatory response to the increase in PGC1α target gene expression, setting the basis for the comprehension of the molecular pathways triggered by Complex I inhibition that may need attention as drawbacks before these approaches are implemented in ovarian cancer care.

## Introduction

Targeting mitochondrial respiratory Complex I (CI), the first enzyme of the oxidative phosphorylation (OXPHOS) system, appears today to be a valid anticancer strategy^1,2^ since the Warburg effect has been revisited to discover that solid tumors may not afford to have a dysfunctional respiratory chain^3^. Several CI inhibitors, among which the widely used antidiabetic drug metformin, are hence currently in clinical trials and efforts are made to synthetize and test a growing number of more specific compounds^4–7^. Nonetheless, we are now increasingly aware that multiple adaptive cell-autonomous and non-cell autonomous responses can be activated in cancer cells to readapt to the loss of a crucial enzyme such as CI^8,9^. Among such compensatory mechanisms, increase in the master regulator of mitochondrial biogenesis, namely Peroxisome Proliferator Activated Receptor Gamma Coactivator 1 alpha (PGC1α), has been shown to occur as a long-term response to CI inactivation following CI genetic ablation^8^ or the occurrence of mitochondrial DNA (mtDNA) disruptive mutations^10^. PGC1α is a pleiotropic transcriptional coactivator whose binding with multiple molecular partners induces gene expression reprogramming crucial during cancer progression, spanning from mitochondrial biogenesis to neoangiogenesis^11,12^. CI inhibition as an anticancer strategy has been proposed, among others, in ovarian cancer (OC), which is one of the most lethal neoplasias, affecting approximately 300,000 women worldwide every year^13^, especially since when symptoms appear, the disease is at advanced stages and often has metastatic spread, particularly at the omentum^14^. Due to the high resistance rates (up to 75% of cases) to standard platinum- and taxane-derived chemotherapies, the introduction of novel potential adjuvant therapies remains an unmet clinical need, and anti-metabolic drugs such as CI inhibitors have recently been proposed as candidates^15^. Although PGC1α has seldom been studied in OC, its activation warrants investigation, as CI inhibitors make their way to clinics. It is known that PGC1α activates fatty acid oxidation^16^, a metabolic route that is necessary for metastatic OC to thrive in the lipid-rich omentum, and binds Estrogen Related Receptor alpha (ERRα), promoting vascularization^17,18^. To foresee potential compensatory mechanisms that may occur upon the use of CI inhibitors in OC patients, we exploited genetic ablation and pharmacological inhibition of this enzyme to identify whether PGC1α may play a role upon the induction of energetic crisis and in the regulation of the metabolic phenotype of OC cells.

## Results

### Levels of PGC1α correlate with mitochondrial abundance in OC cells

**Figure 1:**
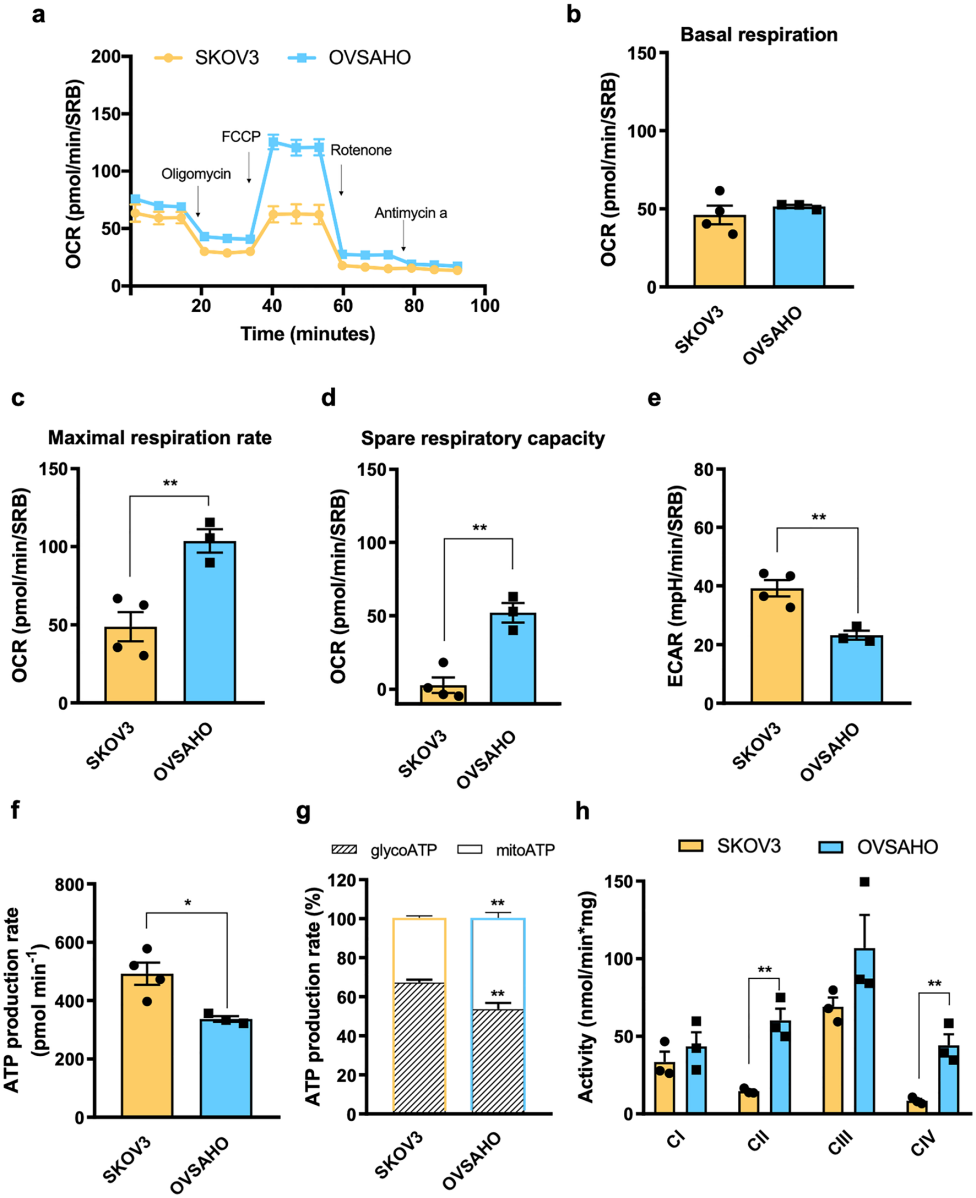
Bioenergetic characterization of high- and low-OXPHOS OC cells. (**a**) Oxygen consumption rate (OCR) profile of SKOV3 (n = 4) and OVSAHO (n = 3) cells determined upon injection of 1 µM oligomycin, 0.5 µM FCCP, 1 µM rotenone and 1 µM antimycin A in Seahorse XFe medium. FCCP concentration was previously determined by titration. Data (mean ± SEM) are normalized on SRB absorbance. (**b**) Basal respiration, (**c**) maximal OCR and (**d**) spare respiratory capacity of SKOV3 (n = 4) and OVSAHO (n = 3) cells. (**e**) Extracellular acidification rate (ECAR) measured under basal conditions. Data (mean ± SEM) are normalized on SRB absorbance. (**f**) Total ATP and (**g**) proportion of mitochondrial ATP (mitoATP) *vs* glycolytic ATP (glycoATP) production rate in SKOV3 (n = 4) and OVSAHO (n = 3) cells measured using Seahorse. Data (mean ± SEM) are normalized to SRB absorbance and the mito/glycoATP ratio is expressed as a percentage of total ATP. (**h**) Spectrophotometric measurement of respiratory complexes activity in SKOV3 (n = 3) and OVSAHO (n = 3) cells. Data (mean ± SEM) are normalized on protein content.

OC is a highly heterogeneous neoplasm for which stratification based on the correlation between metabolism and response to standard chemotherapy allows the identification of low- versus high-OXPHOS tumors^19^. Thus, we assessed the oxygen consumption rate (OCR), the glycolytic indicator extracellular acidification rate (ECAR) and ATP production rate in two different high-grade serous OC cell lines, namely, OVSAHO and SKOV3 to validate in our experimental settings their metabolic features, which had been previously characterized^19^. Despite a comparable basal OCR, the maximal respiration rate of OVSAHO cells was twice that of SKOV3 cells (Fig. 1a–c). Moreover, OVSAHO showed a spare respiratory capacity and reduced ECAR (Fig. 1d,e), suggesting a major contribution of OXPHOS to energy metabolism. Surprisingly, the overall ATP production rate was reduced in OVSAHO cells (Fig. 1f), but this apparent paradox can be explained by the fact that ATP is mainly derived from OXPHOS in this cell model, while SKOV3 cells mostly rely on glycolysis as a source of ATP (Fig. 1g). These data correlated with a trend in increased respiratory complex activities, particularly evident for those of CII and CIV, in OVSAHO cells compared to SKOV3 cells (Fig. 1h). Altogether, these data allowed us to categorize SKOV3 as low-OXPHOS cells with respect to OVSAHO, in our experimental settings, which instead show higher reliance on oxidative metabolism.

**Figure 2:**
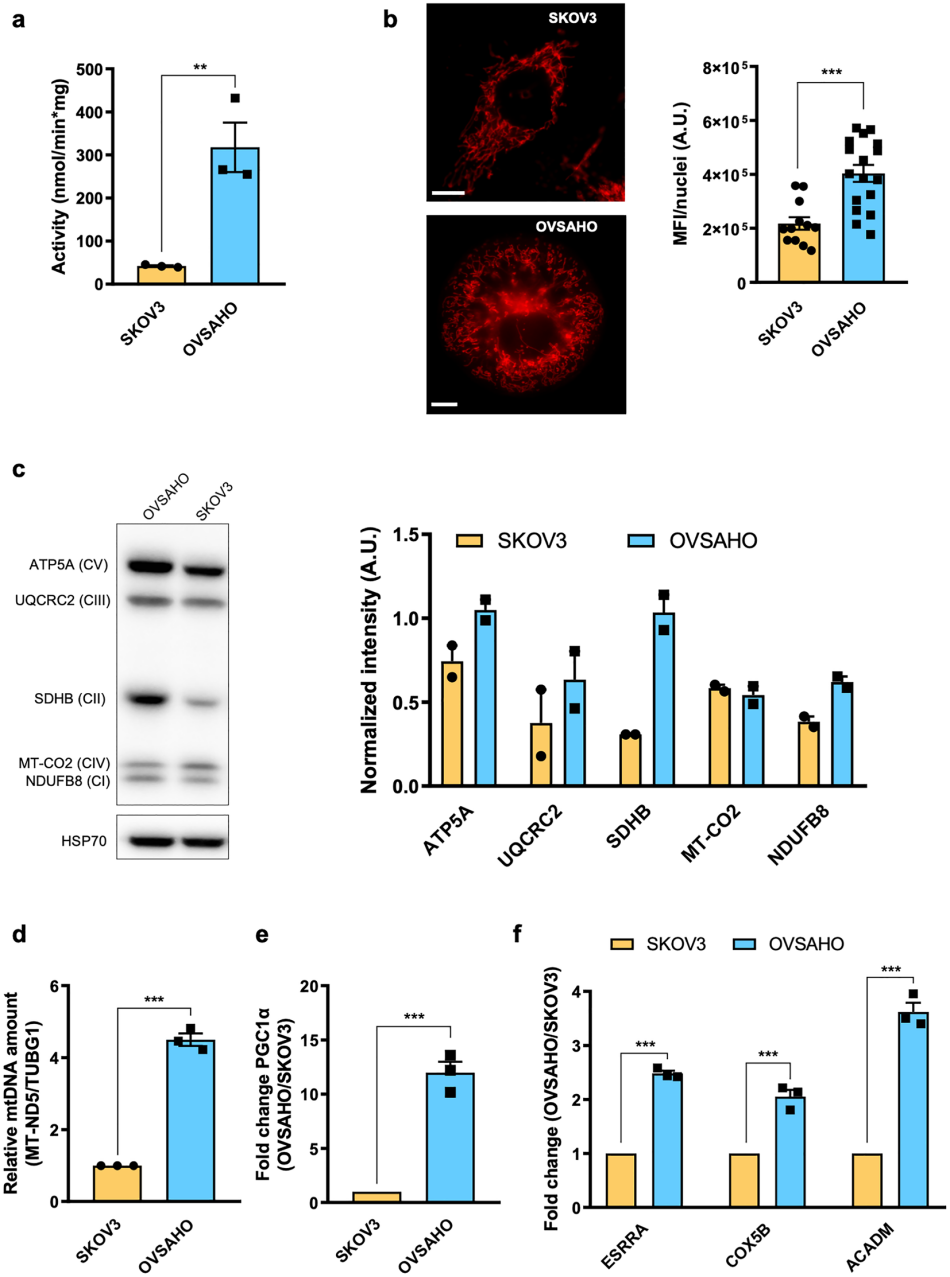
Differential mitochondrial abundance and PGC1⍺ expression in high- and low-OXPHOS OC cells. (**a**) Spectrophotometric measurement of citrate synthase (CS) activity in SKOV3 (n = 3) and OVSAHO (n = 3) cells. Data (mean ± SEM) are normalized on protein content. (**b**) Mitochondrial network evaluated by MitoTracker Red staining in SKOV3 and OVSAHO cells. Representative images are shown. Scale bar represents 10 µm. Mean Fluorescence Intensity (MFI) data (mean ± SEM) were normalized on nuclei number. Original figures are presented in Supplementary Fig. 2a. (**c**) Western blotting analysis of five OXPHOS complex subunits (one for each complex) in SKOV3 (n = 2) and OVSAHO (n = 2) cells. HSP70 was used as loading control. Band intensity was quantified by densitometry. Unnecessary lanes were cropped, and full-length blots are presented in Supplementary Fig. 2b. (**d**) Relative mtDNA amount evaluated by qPCR in SKOV3 (n = 3) and OVSAHO (n = 3) cells. ΔCT = CT (SKOV3)-CT (OVSAHO). Data (mean ± SEM) are expressed as fold change. Relative amount of mitochondrial *MT-ND5* was normalized to *TUBG1*. (**e**) Gene expression of total PGC1α evaluated by qRT–PCR in SKOV3 (n = 3) and OVSAHO (n = 3) cells. ΔCT = CT (SKOV3)-CT (OVSAHO). Data were normalized to the quantity of retrotranscribed total mRNA. (**f**) Gene expression of total *ESRRA*, *COX5B* and *MCAD* in SKOV3 and OVSAHO cells (n = 3). Data are expressed as fold change and represented as the mean ± SEM. GOI (gene of interest); relative expression levels of GOI were normalized to *ACTB* expression for *ESRRA* and *MCAD* and *GUSB* for *COX5B*.

We next investigated whether such divergent metabolic assets may be related to differences in mitochondrial mass and biogenesis. Interestingly, OVSAHO displayed a higher activity of citrate synthase (CS), a well-known indicator of mitochondrial mass, as well as more intense MitoTracker Red staining (Fig. 2a,b). Moreover, the steady-state levels of most of the analyzed OXPHOS complex subunits were higher in OVSAHO cells (Fig. 2c), along with the relative mitochondrial DNA (mtDNA) abundance, which was indeed nearly 5-fold higher in OVSAHO than in SKOV3 cells (Fig. 2d). Overall, these data indicate a richer mitochondrial phenotype, possibly due to a more active mitochondrial biogenesis in OVSAHO cells compared to SKOV3 cells. The master regulator of mitochondrial biogenesis is the transcriptional coactivator PGC1α, which exerts pleiotropic transcriptional control of several downstream pathways. Hence, we investigated PGC1α gene expression and that of some of its responsive genes specifically selected to be representative of such diverse pathways in the two OC cell models. We analyzed the levels of *COX5B,* coding for cytochrome *c* oxidase subunit Vb and thus representative of OXPHOS, *ESRRA*, which encodes the Estrogen Related Receptor α (ERRα), a transcription factor known to regulate energy metabolism^20^ and *ACADM*, producing medium-chain acyl-CoA dehydrogenase (MCAD*),* crucial for lipid metabolism, the main source of energy for OC omental metastases^21–23^. In agreement with their high OXPHOS status and the more abundant mitochondrial mass, OVSAHO showed a 12-fold higher expression of PGC1α and a significant increase in all its analyzed responsive genes compared to SKOV3 (Fig. 2e,f), suggesting that their elevated OXPHOS status may derive from upregulated mitochondrial biogenesis with a concurrent activation of other pathways regulated by PGC1α.

### Genetic ablation of CI triggers PGC1α expression and activation under glucose restriction

**Figure 3:**
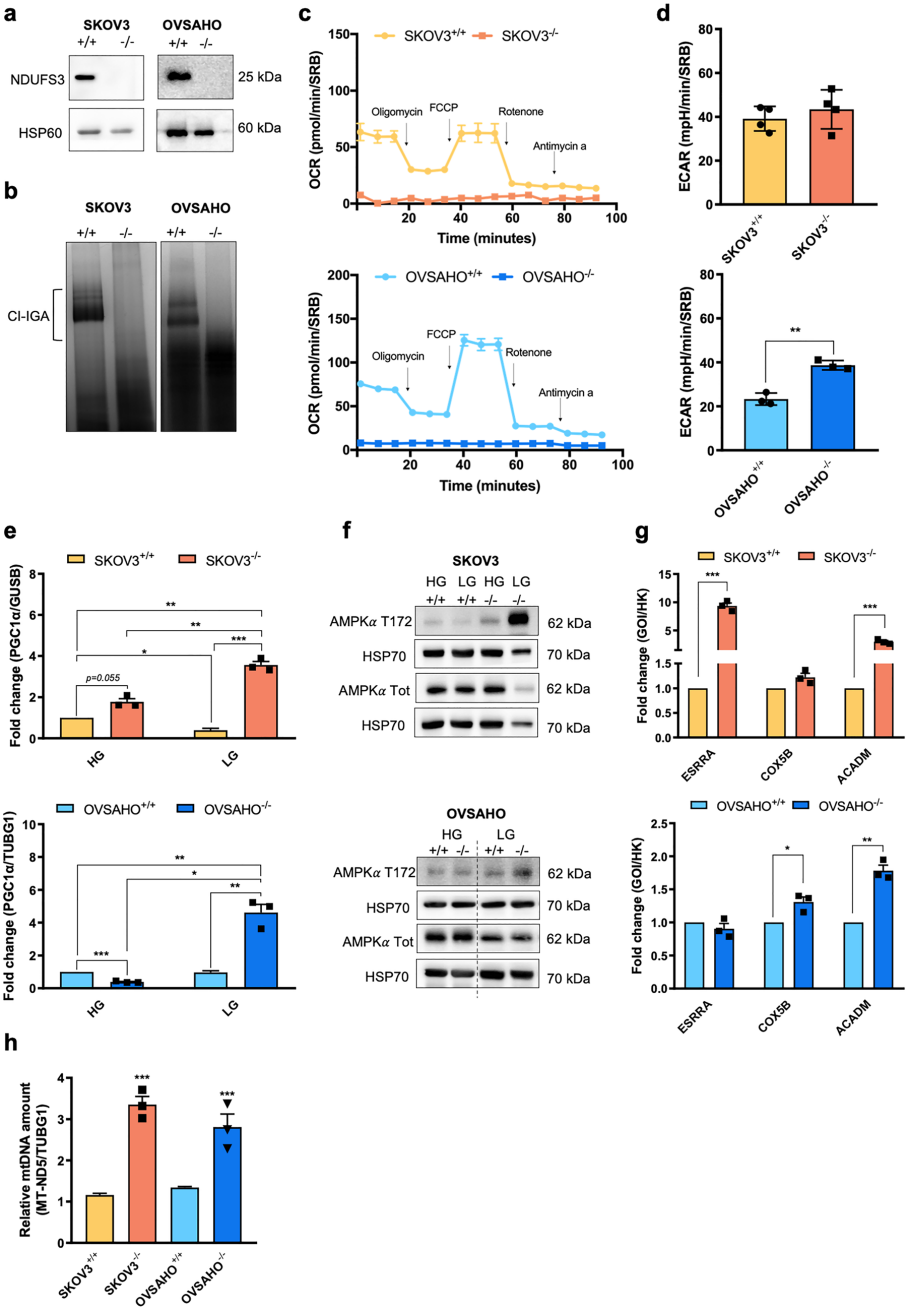
PGC1α expression increases in CI-null OC cell lines upon glucose restriction. (**a**) NDUFS3 western blotting analysis of mitochondrial-enriched fractions from SKOV3^+/+^, SKOV3^−/−^, OVSAHO^+/+^ and OVSAHO^−/−^ cell lines separated by SDS–PAGE. HSP60 was used as loading control. Unnecessary lanes were cropped. Full-length blots are presented in Supplementary Fig. 3a,b (**b**) CI *in-gel* activity (CI-IGA) of mitochondrial enriched fractions from SKOV3^+/+^, SKOV3^−/−^, OVSAHO^+/+^ and OVSAHO^−/−^ samples solubilized with DDM and separated by BN-PAGE. Unnecessary lanes were cropped. Full-length gels are presented in Supplementary Fig. 3c. (**c**) Oxygen consumption rate (OCR) profile of SKOV3^+/+^ (n = 4), SKOV3^−/−^ (n = 4), OVSAHO^+/+^ (n = 3) and OVSAHO^−/−^ (n = 3) cells determined upon injection of 1 µM oligomycin, 0.5 µM FCCP, 1 µM rotenone and 1 µM antimycin A in specific Seahorse XFe medium. FCCP concentration was determined by titration. Data (mean ± SEM) are normalized on SRB absorbance. (**d**) Extracellular acidification rate (ECAR) measured under basal conditions of SKOV3^+/+^ (n = 4), SKOV3^−/−^ (n = 4), OVSAHO^+/+^ (n = 3) and OVSAHO^−/−^ (n = 3) cells. Data (mean ± SEM) are normalized on SRB absorbance. (**e**) PGC1α expression in SKOV3^+/+^ (n = 3), SKOV3^−/−^ (n = 3), OVSAHO^+/+^ (n = 3) and OVSAHO^−/−^ (n = 3) cells grown in 25 mM (HG) or 5 mM (LG) glucose for 24 h. Data are expressed as fold change and normalized to relative PGC1α levels in NDUFS3^+/+^ models grown in HG. (**f**) Western blotting analysis of phosphorylated (T172) and total AMPKα levels on SKOV3^+/+^, SKOV3^−/−^, OVSAHO^+/+^ and OVSAHO^−/−^ cellular lysates under 25 mM (HG) and 5 mM (LG) glucose growth conditions. HSP70 was used as loading control. The dotted line indicates non-contiguous lanes deriving from the same gel and exposure. Full-length blots are presented in Supplementary Fig. 4a,b. The intensity of each band was quantified by densitometry and data (mean ± SEM) were expressed as fold of phosphorylated (T172) to total AMPKα (Supplementary Fig. 1a). (**g**) Gene expression of total *ESRRA*, *COX5B* and *ACADM* evaluated by qRT–PCR in SKOV3 and OVSAHO cells grown in LG for 24 h (n = 3). Data are expressed as fold change and represented as the mean ± SEM. GOI (gene of interest); HK (housekeeping gene); relative expression levels of GOIs are calculated on *ACTB* expression for *ESRRA* and *ACADM* and *GUSB* for *COX5B*. (**h**) Relative mtDNA amount evaluated by qPCR in SKOV3^+/+^, SKOV3^−/−^, OVSAHO^+/+^ and OVSAHO^−/−^ cells grown in HG or LG for 24 h (n = 3). ΔCT = CT (LG)-CT (HG). Data (mean ± SEM) are expressed as fold change. Relative amount of mitochondrial *MT-ND5* was normalized to *TUBG1*.

We next proceeded to gauge whether a potent stimulus such as CI derangement may induce changes in PGC1α expression in OC cells like those observed in other cancer contexts, such as osteosarcoma^8^. To this aim, we generated syngenic cell lines devoid of the core CI subunit *NDUFS3 via* gene editing (SKOV3^−/−^ and OVSAHO^−/−^), in which protein expression was completely abolished (Fig. 3a). Similar to our previous findings in other cell lines^8^, the lack of NDUFS3 induced almost complete CI disassembly, causing its dysfunction (Fig. 3b) and the consequent abolishment of mitochondrial respiration (Fig. 3c). Interestingly, while ECAR was unaffected by the lack of CI in glycolytic low-OXPHOS SKOV3 cells, its values increased when *NDUFS3* was ablated in oxidative OVSAHO cells (Fig. 3d), suggesting the occurrence of an adaptive metabolic switch toward glycolysis in the latter model. Then, we measured PGC1α levels in resting conditions (25 mM glucose - high glucose; HG), in which no increase was observed between CI-null and CI-competent cells (Fig. 3e), in contrast to what we previously found in osteosarcoma^8^. We reasoned that OC cells may not respond by increasing PGC1α upon CI dysfunction if a metabolic stress is not induced. To this aim, we cultured cells under glucose deprivation (5 mM glucose-low glucose; LG) for 24 hours, which induced an energetic impairment in CI-null cells, as testified by the activation of the main energy sensor AMP activated kinase (AMPK) (Fig. 3f and Supplementary Fig. 1a). Under these conditions, a significant increase in PGC1α expression was observed in CI-null cells compared to their CI-competent counterparts in both OVSAHO and SKOV3 background (Fig. 3e), which was followed by the upregulation of two out of three PGC1α-responsive genes tested (Fig. 3g). Interestingly, the PGC1α increase upon energetic impairment was more prominent in SKOV3 than in OVSAHO cells (10- versus 5-fold, respectively; Fig. 3e and Supplementary Fig. 1b), likely since the former needs to foster mitochondrial biogenesis to efficiently reprogram metabolism, whereas the latter intrinsically has a higher mitochondrial respiration. To gauge whether the increase in PGC1α translated into an augmented mitochondrial biogenesis, we evaluated mtDNA copy number and revealed approximately a 3-fold increase in LG in both CI-null cell lines, suggesting this response was independent from the cell metabolic features (Fig. 3h). Overall, these data demonstrate that the energy crisis caused by glucose restriction in synergy with CI ablation triggers a PGC1α-mediated compensatory response regardless of the OXPHOS status of the cells.

### Pharmacological inhibition of CI mimics its derangement and induces PGC1α compensatory expression and activation

**Figure 4:**
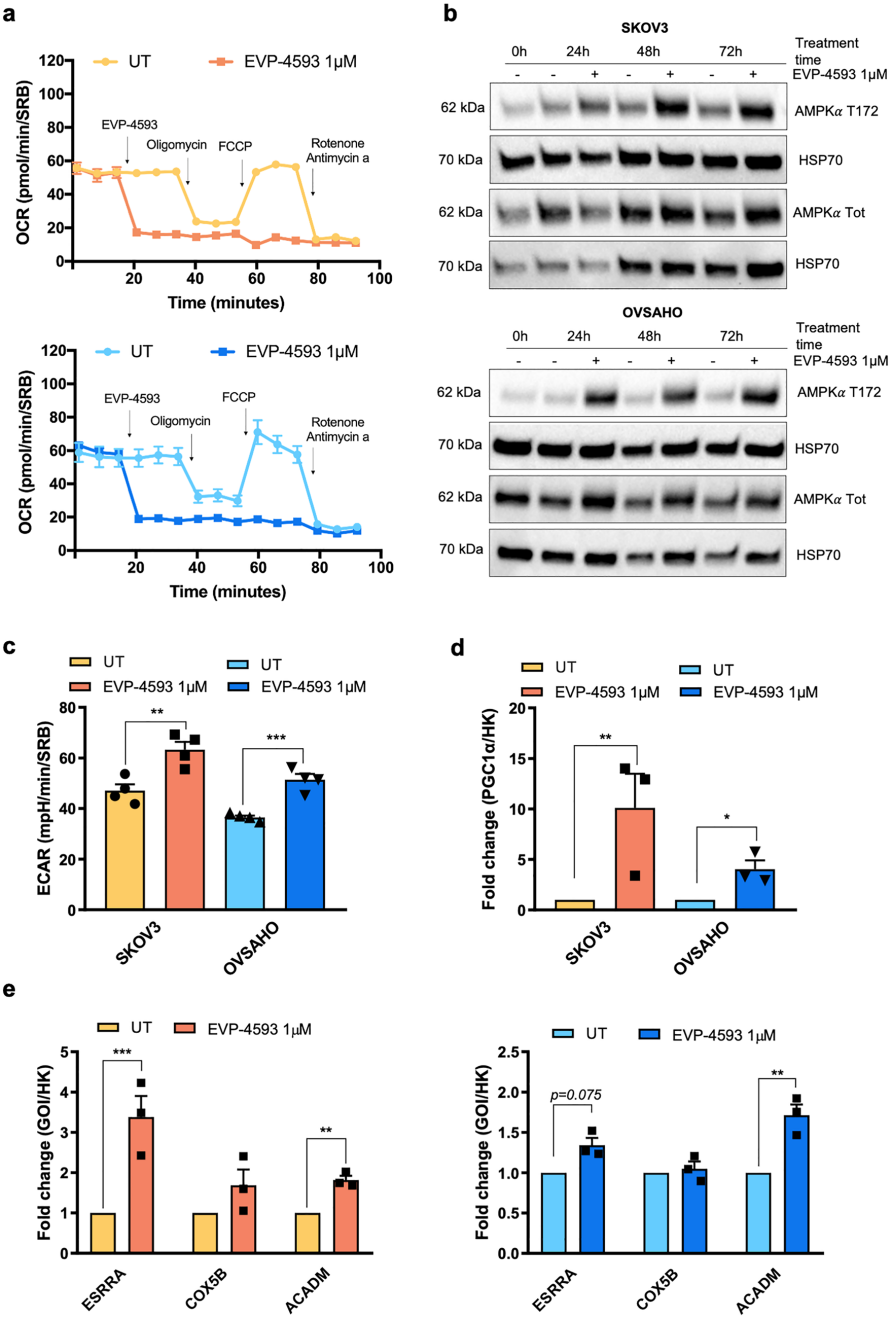
CI inhibition recapitulates the compensatory PGC1α upregulation under energetic crisis found in CI-null models. (**a**) Oxygen consumption rate (OCR) profile of SKOV3 (n = 2) cells determined upon injection of 1 µM EVP-4593, 1 µM oligomycin, 0.5 µM FCCP, 1 µM rotenone and 1 µM antimycin A in 5 mM glucose (LG) Seahorse XFe medium. FCCP concentration was determined by titration. Untreated (UT) samples received the injection of EVP-4593 solvent DMSO. Data (mean ± SEM) are normalized on SRB absorbance. (**b**) Western blotting analysis of phosphorylated (T172) and total AMPKα levels in lysates from SKOV3 and OVSAHO cells untreated and treated with 1 µM EVP-4593 at different time points and cultured in LG. HSP70 was used as loading control. Full-length blots are presented in Supplementary Fig. 5a,b. Densitometric analysis is shown in Supplementary Fig. 1c. (**c**) Extracellular acidification rate (ECAR) measured in LG and upon treatment with 1 µM EVP-4593 in SKOV3 and OVSAHO cells (n = 4). Data (mean ± SEM) are normalized on SRB absorbance. (**d**) Gene expression of total PGC1α in SKOV3 and OVSAHO cells treated with 1 µM EVP-4593 (n = 3) cultured in LG. (**e**) Gene expression of *ESRRA*, *COX5B* and *ACADM* in SKOV3 and OVSAHO cells after treatment with 1 µM EVP-4593 (n = 3). Cells were cultured in LG. Data are expressed as fold change and represented as the mean ± SEM. GOI (gene of interest); HK (housekeeping gene); relative expression levels of GOIs are calculated on *ACTB* expression for *ESRRA* and *ACADM* and *GUSB* for *COX5B.*

Last, with the intent to test whether the mechanisms displayed by cells in which CI was genetically ablated were adaptive or generally activated when CI was pharmacologically inhibited, we exploited the recently synthesized selective CI inhibitor EVP-4593, which is known to bind the ubiquinone reduction site of CI and has been preliminarily tested as an anticancer molecule^24^. First, we found that 1 µM EVP-4593 was able to completely abolish mitochondrial respiration in both SKOV3 and OVSAHO cells (Fig. 4a). This was associated with a marked increase in AMPK phosphorylation at Thr172 starting from 24 h for OVSAHO and 48 h for SKOV3 when cells were grown in LG, suggesting that the energy crisis had occurred (Fig. 4b and Supplementary Fig. 1c). Upon inhibition of CI with 1 µM EVP-4593 in LG, ECAR was increased in both cell lines (Fig. 4c), corroborating the idea that a metabolic switch toward glycolysis may occur. In agreement and similar to what was observed in genetically ablated SKOV3^−/−^ and OVSAHO^−/−^ cells, CI inhibition by 1 µM EVP-4593 in LG induced a 10-fold increase in PGC1α expression in SKOV3 cells and nearly 5-fold in OVSAHO cells (Fig. 4d). Moreover, in both cell lines, the treatment caused a rise in the expression of two out of three PGC1α-responsive genes (Fig. 4e). Taken together, pharmacological targeting of CI triggered a PGC1α-mediated compensatory mechanism, recapitulating the phenomenon described in NDUFS3 knockout cells and suggesting that this was both an acute and an adaptive response.

## Discussion

In this work, we set the basis for the study of the pleiotropic transcriptional coactivator PGC1α in OC. In particular, we correlated higher PGC1α expression and activity with higher OXPHOS metabolic status in OC cells, indicating that this coactivator is involved in the maintenance of mitochondrial mass required for elevated respiration and may be used as a marker when defining the OXPHOS-related metabolic status of OC cells. In this context, it is interesting to note that, compared to previous findings^19^, OVSAHO cells here are characterized by higher levels of mitochondrial proteins, enzymes activity and maximal respiration compared to SKOV3 cells, suggesting they exhibit a higher OXPHOS metabolism, and prompting that plastic parameters such as those defining cell bioenergetics should be evaluated prior to any investigation. Moreover, we investigated the PGC1α-mediated response to genetic or pharmacologic respiratory CI inactivation in OC cell models. A comprehension of the PGC1α response to pharmacological stimuli is required in cancer, since it has been defined as an *oncojanus* gene^25^, whereby it behaves as an oncogene^26,27^ or as a tumor suppressor gene^28^ according to the context. PGC1α is widely recognized as the master regulator of mitochondrial biogenesis and thus controls oxidative metabolism by promoting the expression of OXPHOS complexes^29^. In this frame, PGC1α has been shown to contribute to the metabolic heterogeneity of different types of human cancers and to modulate their response to chemotherapy^30–33^. Moreover, since the discovery that the interaction with ERRα renders PGC1α a regulator of hypoxia-independent vasculogenesis *via* transcription of the Vascular Endothelial Growth Factor (VEGF)^17^, more attention has been paid to PGC1α in cancer. However, much remains to be unveiled on what stimuli are able to trigger a process that may importantly impinge on tumor progression toward malignancy. This is of relevance in OC, where ERRα is highly expressed^34^, and vascularization is a cogent issue so that the anti-VEGF drug bevacizumab has been introduced, not without severe side effects, in the therapeutic regimen of advanced cases along with standard therapy^35^. An increase in PGC1α was previously shown to occur following CI genetic ablation or in the presence of disruptive mtDNA mutations^8,10^. We show here that inhibition of CI triggers a compensatory response increasing PGC1α expression, which has not been shown thus far. Indeed, a pharmacological approach is quickly emerging as an effective anticancer therapy based on the notion that cancer cells must rely on a functional respiratory chain to thrive^36^. The most plausible mechanism explaining the PGC1α increase upon CI inhibition may be retrograde signaling triggered by mitochondrial energetic impairment, which was previously shown to be mediated by AMPK^37^. In particular, AMPK activates PGC1α at post-translational level inducing its expression in a reinforced positive feedback loop^37^. The increase in PGC1α, albeit at different levels, occurs in both high- and low-OXPHOS OC cells, indicating that CI inhibition is a potent stimulus for PGC1α increase regardless of the intrinsic oxidative capacity of the tumor. It is important to note that apart from its regulation of mitochondrial biogenesis, PGC1α has been associated with several other processes relevant in the context of cancer. Indeed, we report that an increase in PGC1α is associated with augmented levels of specific PGC1α target genes involved in different pathways, such as mitochondrial energy metabolism, angiogenesis and lipid catabolism. Interestingly, while *COX5B* does not appear to increase upon an acute stimulus, suggesting that a trigger of mitochondrial biogenesis requires coordination with the mitochondrial genome and may rather be an adaptive response, *ESRRA* is among the PGC1α-responsive genes to be upregulated. It is plausible that strong consequent angiogenesis may be fostered, therefore, by a joint increase in both constituents of the molecular complex responsible for VEGF transcription^17^, calling for attention when using CI inhibitors in OC. *MCAD* was also shown to increase in association with PGC1α and CI inhibition; this is also relevant in OC, as it is well known that omental metastases preferentially use lipid catabolism, exploiting the lipid-rich environment by producing fatty acid binding protein 4 to internalize fatty acids from adipocytes^38,39^.

In conclusion, our data begin to unravel the PGC1α-mediated compensatory responses that may be triggered in OC when CI is hampered as a therapeutic approach. As PGC1α increase occurs independently of the initial and basal metabolic status of the cell, CI inhibition may be a strong and horizontal strategy to induce an energy crisis in both high- and low-OXPHOS cancer cells, but its implementation warrants investigation to counteract or prevent adaptive responses.

## Methods

### Cell lines and treatments

The human ovarian cancer cell lines SKOV3 and OVSAHO were purchased from ATCC^®^ (Manassas, VA, USA) and JCRB Cell Bank (Japan), respectively. Cells were cultured in High Glucose Dulbecco's Modified Eagle's Medium (DMEM) with sodium pyruvate (Euroclone #ECB7501L) supplemented with 10% FBS South America origin EU Approved (Euroclone #ECS5000L), 2 mM L-Glutamine (Euroclone #ECB3000D), 1% Penicillin/Streptomycin (Euroclone #ECB3001D) and 50 µg/mL uridine (Sigma–Aldrich #U3003) and maintained at 37 °C in a humidified atmosphere with 5% CO_2_. For high- or low-glucose experiments, cells were grown for 24 hours in DMEM (Gibco #11966025) supplemented with 10% FBS, 2 mM L-glutamine, 1% penicillin/streptomycin, 50 µg/mL uridine, 1 mM sodium pyruvate (Sigma–Aldrich #P2256) and 25 mM or 5 mM D-(+)-glucose (Sigma Aldrich #G7021). Where indicated, cells were treated with 1 µM N4-[2-(4-Phenoxyphenyl) ethyl]-4,6-quinazolinediamine [EVP-4593; Sigma–Aldrich #SML0579] at multiple timepoints (6 h, 12 h, 24 h) and compared to Time 0 (T0). EVOS M5000 Imaging System (Thermo Fisher Scientific #AMF5000) was used for cell line monitoring.

### Genome Editing

The CRISPR/Cas9 system was used to insert a frameshift mutation in the NDUFS3 gene in SKOV3 and OVSAHO cell lines. Cas9 protein was transfected following the manufacturer’s instructions using Lipofectamine CRISPRMAX Cas9 Transfection reagent (Invitrogen #CMAX00008) together with synthetic RNA guides designed and purchased from IDT. Exon 2 targeting guide TGTCAGACCACGGAATGATG was used. Non-homologous repair efficiency was evaluated by Sanger sequencing using KAPA2G Taq Polymerase (Kapa Biosystems #KK5601) and the Big Dye protocol (Life Technologies #4337451). PCR for NDUSF3 was performed using the primers forward 5’-TCTCAAGGTGCTTCAGGGAG-3’ and reverse 5’-GAAACAAGTCTGCCCACTCC-3’. Clonal selection was carried out to select cells with frameshift NDUFS3 mutations. DNA extraction was performed following the manufacturer’s instructions using 8 µL of lysis buffer (Sigma–Aldrich #L3289) and 80 µL of neutralization buffer (Sigma Aldrich #N97784) per sample in a 96-well plate.

### Gene expression quantitative real-time PCR

SKOV3 (2 × 10^5^ cells) and OVSAHO (4.5 × 10^5^ cells) cells were seeded in a 6-well plate and grown in 25 or 5 mM glucose for 24 hours. RNA was extracted from cell pellets using RNeasy mini kit (QIAGEN #74106) and quantified by NanoDrop^TM^ 2000 Spectrophotometer (Thermo Scientific). Three hundred nanograms of total RNA was reverse-transcribed into cDNA using the High-Capacity cDNA Reverse Transcription Kit (Applied Biosystems #4368814) with random hexamers. Quantitative real-time PCR (qRT–PCR) was performed using either the intercalating dye SYBR^®^ Green dye (Promega) or 5’ nuclease probes PrimeTime™ qPCR Probes (TaqMan assay). For the SYBR Green assay, the primer sequences were designed using Primer3 software^40^. The presence of 3’ intra/inter primer homology was excluded using the IDT OligAnalyzer tool (https://eu.idtdna.com/analyzer/Applications/OligoAnalyzer/), and the availability of the target sequence was estimated by predicting cDNA secondary structure by the Mfold web server^41^. For TaqMan assays, the PrimeTime™ qPCR Probes assay for each gene was selected on the IDT website (https://eu.idtdna.com/site/order/qpcr/predesignedassay), and the predesigned qPCR assay recommended by the manufacturer was used. qRT–PCR with SYBR Green assay was performed with GoTaq qPCR Master Mix (Promega #A6002) and run in 7500 Fast Real-Time PCR System (Applied Biosystem), using the following conditions: 95 °C 5 min; 45 cycles of 95 °C 15 s and 63 °C 45 s. The qRT–PCR with TaqMan assay was performed with GoTaq^®^ Probe qPCR Master Mix (Promega #A6101 and #A6102) and run in the abovementioned system, using the following conditions: 95 °C 2 min; 40 cycles of 95 °C 15 s and 60 °C 1 min. Unless stated otherwise, the analysis was performed using the 2^−ΔΔCT^ method [CT (control)-CT (experiment)], where the control was calculated as the average CT value obtained from control samples. For the SYBR Green assay, the following housekeeping genes were used: *GUSB* for SKOV3 and *TUBG1* for OVSAHO. For the TaqMan assay, the housekeeping gene ACTB was used for both cell lines. The statistical significance was calculated using ΔCT values [CT (gene of interest)-CT (reference gene)] for each biological replicate^42^. Primer sequences for SYBR and TaqMan assays can be found in Supplementary Table 1-2.

### MtDNA relative amount evaluation

SKOV3 (2 × 10^5^ cells) and OVSAHO (4.5 × 10^5^ cells) cells were seeded in a 6-well plate and grown in 25 or 5 mM glucose for 24 hours. DNA was extracted from cell pellets with QIAamp DNA Blood Mini kit (QIAGEN #51106) and quantified by NanoDrop^TM^ 2000 Spectrophotometer (Thermo Scientific). qPCR was performed using 5 ng of DNA with primers recognizing *MT-ND5* (forward: 5’-ATCCTTCTTGCTCATCAGTTG-3’, reverse: 5’-GGCTATTTGTTGTGGGTCTC-3’) for mtDNA detection and *TUBG1* (forward: 5’-CCCTGGCTACATGAACAATG-3’, reverse: 5’-GTAGCCGGTCATGAGGAAGT-3’) as nuclear housekeeping gene. The amplification reaction was set up with GoTaq qPCR Master Mix (Promega #A6002) and run in 7500 Fast Real-Time PCR System (Applied Biosystem), using the following conditions: 95 °C 10 min; 40 cycles of 95 °C 15 s and 60 °C 45 s. The statistical significance was calculated using ΔCT values [CT (*MT-ND5*)-CT (*TUBG1*)] for each replicate^42^.

### Microrespirometry and extracellular acidification rate assessment

Oxygen consumption rate (OCR) and extracellular acidification rate (ECAR) were measured using the protocol described for the Seahorse XFe Cell Mito Stress Test Kit (Agilent #103015-100) following the manufacturer’s instructions. A total of 1.2 × 10^4^ cells/well (SKOV3) and 2 × 10^4^ (OVSAHO) were seeded in 80 μL of DMEM into XFe96 cell culture plates and incubated for 24 hours at 37 °C and 5% CO_2_. Seeding was optimized before the assay. Complete growth medium was replaced with 180 μL of XFe medium (Agilent #103575-100) supplemented with 10 mM glucose, 1 mM sodium pyruvate, and 2 mM L-glutamine at pH 7.4. For temperature and pH equilibration, cells were incubated at 37 °C for 30 min. After three OCR baseline measurements, 1 μM oligomycin, 0.5 μM carbonyl cyanide-p-trifluoromethoxyphenylhydrazone (FCCP), 1 μM rotenone, and 1 μM antimycin A were sequentially added to each well. In the case of inhibition with 1 µM EVP-4593, rotenone and antimycin A were added together. FCCP concentrations were optimized in each cell line by titration before the experiments. At the end of the assay, the medium was removed, and a sulforhodamine B (SRB) assay was performed to determine the protein content. Briefly, plates were incubated with 10% trichloroacetic acid (TCA) for 1 h at 4 °C to fix the cells. Five washes in water were carried out. Once the plates were dried, proteins were stained by incubation with 0.4% SRB for 30 min at RT. Then, SRB was solubilized with 10 mM Tris, and the absorbance at 560 nm was determined using a Victor2 plate reader (Perkin-Elmer). Each biological replicate experiment (n=3-4) included measurements from at least six wells. Data (pmol/min) were normalized to blank corrected SRB absorbance. ATP production rate was determined using the protocol described for the Seahorse XF Real-Time ATP Rate Assay Kit (Agilent #103592-100).

### Mitochondrial fraction preparation

Mitochondrial-enriched fractions were obtained by subcellular fractionation (10 × 10^6^ cells/mL) in the presence of 50 µg/mL digitonin (Calbiochem, #3000410). These samples were used for SDS–PAGE and Blue Native PAGE (BN-PAGE) experiments. Crude mitochondria were isolated from 20 to 40 × 10^6^ cells, suspended in sucrose-mannitol buffer (200 mM mannitol, 70 mM sucrose, 1 mM EGTA and 10 mM Tris-HCl at pH 7.6) and homogenized using a glass/Teflon Potter-Elvehjem homogenizer. Differential centrifugation (600 g for 10 min at 4 °C followed by 10,000 g for 20 min at 4 °C) was performed to separate crude mitochondria from other subcellular fractions. The resulting pellets were resuspended in sucrose-mannitol buffer, stored at − 80 °C and used for spectrophotometric determination of respiratory complexes and citrate synthase (CS) activity.

### Spectrophotometric kinetic assays of enzymatic activities

CI (NADH/dichlorophenol indophenol (DCIP) oxidoreductase, rotenone sensitive), CII (succinate/DCIP oxidoreductase, malonate sensitive), CIII (cytochrome *c*/decylubiquinol oxidoreductase, antimycin A sensitive), and CIV (cytochrome *c* oxidase, potassium cyanide sensitive) activities were measured on crude mitochondria in a spectrophotometer (V550 Jasco Europe, Italy) at 37 °C as previously described^43^. For citrate synthase activity, mitochondria were incubated with 100 µM dithionitrobenzoic acid (DTNB), 300 µM acetyl-CoA, 500 µM oxalacetate, 100 mM Tris (pH 8.1) and 0.1% Triton X-100^43^. Data (n=3) were normalized to protein concentration and expressed as nmol/min x mg.

### Mitochondrial network staining

Cells (1 × 10^5^ cells/dish) were seeded on glass cover slides (Ø 10 mm) and incubated with 2 mL of culture medium. After 24 hours, the cells were incubated with 10 nM MitoTracker Red CMXRos (Invitrogen, #M7512) for 10 min at 37 °C. After incubation, the cells were washed with PBS, and the slide was placed in a specific metal grid with 1 mL of DMEM without red phenol supplemented with 25 mM HEPES (Gibco #21063029). The mitochondrial reticulum was visualized with a digital imaging system using an inverted epifluorescence microscope with a ×63/1.4 numerical aperture (NA) oil objective (Nikon Eclipse T*i*-U; Nikon). Images were captured with a back-illuminated Photometrics Cascade CCD camera system (Roper Scientific) and elaborated with Metamorph Acquisition/Analysis Software (Universal Imaging Corp.). Fluorescence intensity analysis was performed using ImageJ^44^. Fluorescence intensity data for each image were normalized to the nuclei number.

### SDS–PAGE and Western Blot

Whole lysates of cultured cells were prepared in RIPA buffer (50 mMTris–HCl pH 7.4, 150 mM NaCl, 1% SDS, 1% Triton, 1 mM EDTA pH 7.6) supplemented with inhibitors of proteases (Thermo Scientific #A32955) and phosphatases (Thermo Scientific #A32957) and quantified using *DC* protein assay (Bio–Rad #5000116). For immunodetection of NDUFS3, lysates from mitochondrial-enriched fractions were used. Samples were separated by SDS–PAGE using a TGX^TM^ FastCast^TM^ Acrylamide kit, 10% (Bio–Rad #1610173), and transferred onto nitrocellulose membranes using a Turbo-pack system (Bio–Rad #1704159SP5). Membranes were blocked at 37 °C for 30 min and incubated with primary antibodies using the following conditions and dilutions: anti-AMPKα (Cell Signaling Technology, #2532) 1:1000 overnight at 4 °C, anti-phospho-AMPKα (Thr172) (40H9) (Cell Signaling Technology, #2535) 1:1000 overnight at 4 °C, anti-HSP60 (Santa Cruz Biotechnology, #sc-13966) 1:1000 for 1 h at RT, anti-HSP70 (BD Transduction, #H53220) 1:1000 for 1 h at RT, anti-total OXPHOS (Abcam, ab110411), anti-NDUFS3 (Abcam, #110246) 1:1000 overnight at 4 °C. Membranes were washed using TBS-Tween (0.1% Tween 20 (Sigma–Aldrich, #P9416) in Tris-buffered saline). Secondary antibodies (Jackson ImmunoResearch Laboratories, #111035144 and #111035146) were incubated for 30 min at RT using 1:20000 and 1:10000 dilutions in TBS-Tween for anti-mouse and anti-rabbit secondary antibodies, respectively. Membranes were developed using Clarity Western ECL Substrate (Bio–Rad #1705061), and ECL was detected with ChemiDoc (Bio–Rad). Densitometric analysis was performed using ImageJ^44^.

### Complex I In-Gel Activity (CI-IGA)

Complex I was separated in its native form by blue-native PAGE^45^. Mitochondrial-enriched fractions were solubilized in 1.5 M aminocaproic acid and 50 mM Bis-Tris/HCl at pH 7 with the addition of 2.5 μg *n*-dodecyl β-D-maltoside (DDM)/μg mitochondrial proteins (Sigma–Aldrich, #D4641). Suspensions were incubated at 4 °C for 5 min and centrifuged at 18,000 g and 4 °C for 30 min. Sample buffer (750 mM aminocaproic acid, 50 mM Bis-Tris/HCl at pH 7, 0.5 mM EDTA and 5% Coomassie Brilliant Blue G250) was added, and 30 μg of protein was loaded on 3-12% native PAGE gradient gels at 150 V and 4 °C for *ca.* 3 h. Cathode Buffer A (50 mM tricine, 7.5 mM imidazole, 0.002% Coomassie Brilliant Blue G250, pH 7), Cathode Buffer B (50 mM tricine, 7.5 mM imidazole, pH 7), and Anode Buffer (25 mM imidazole, pH 7) were used. Cathode A was replaced with Cathode B when the frontline was halfway of the gel. Finally, BN-PAGE gels were incubated with a CI-IGA solution (0.1 mg/mL NADH, 2.5 mg/mL 3-(4,5-dimethylthiazol-2-yl)-2,5-diphenyltetrazolium bromide (MTT), 0.5 M Tris/HCl, pH 7.4) at room temperature for 15 min.

### Statistical analysis

Statistical analyses were performed using GraphPad Prism v.8 (GraphPad Software Inc., San Diego, CA, USA). Data were expressed as mean±SEM. Unless stated otherwise, a two-tailed unpaired Student’s t tests assuming equal variances was performed and at least three biological replicates were conducted for each experiment. Statistical significance was defined by *P* value <0.05.

## Supplementary Information

Supplementary Information.
