# Supplementary material for: Inducing respiratory complex I impairment elicits an increase in PGC1α in ovarian cancer
Source: Sci Rep. 2022 May 16;12:8020. doi: 10.1038/s41598-022-11620-y (PMC9110394; doi:10.1038/s41598-022-11620-y)
Supplement: Supplementary file 1 — Supplementary Information. [file 41598_2022_11620_MOESM1_ESM.pdf]

Supplementary Information

Inducing respiratory Complex I impairment elicits an increase in PGC1α in ovarian cancer

Monica De Luise[1,2,3,+], Manuela Sollazzo[2,3,4,+], Eleonora Lama[1,2,3,+], Camelia Alexandra Coada[1,2,3], Licia Bressi[1,2,3], Maria Iorio[1,2,3], Beatrice Cavina[1,2,3], Luigi D'Angelo[2,3,4], Sara Milioni[1,2,3], Lorena Marchio[1,2,3], Stefano Miglietta[2,3,4], Sara Coluccelli[1,2,3], Greta Tedesco[1,2,3], Anna Ghelli[2,3,4], Silvia Lemma[1,2,3], Anna Myriam Perrone[1,3,5], Ivana Kurelac[1,2,3,*], Luisa Iommarini[2,3,4,*], Anna Maria Porcelli[2,3,4,6,‡] and Giuseppe Gasparre[1,2,3,*,‡]

[1]Department of Medical and Surgical Sciences (DIMEC), University of Bologna, Bologna, 40138, Italy
[2]Center for Applied Biomedical Research, University of Bologna, Bologna, 40138, Italy
[3]Centro Studi e Ricerca sulle Neoplasie Ginecologiche (CSR), University of Bologna, Bologna, 40138, Italy
[4]Department of Pharmacy and Biotechnology (FABIT), University of Bologna, Bologna, 40126, Italy
[5]Division of Oncologic Gynecology, IRCCS Azienda Ospedaliero-Universitaria di Bologna, 40138 Bologna, Italy
[6]Interdepartmental Center of Industrial Research (CIRI) Life Science and Health Technologies, University of Bologna, Ozzano dell'Emilia, 40064, Italy

*ivana.kurelac@unibo.it; luisa.iommarini2@unibo.it; giuseppe.gasparre3@unibo.it
[+]these authors contributed equally to this work
[‡]these authors share equal senior authorship

Supplementary Fig. 1

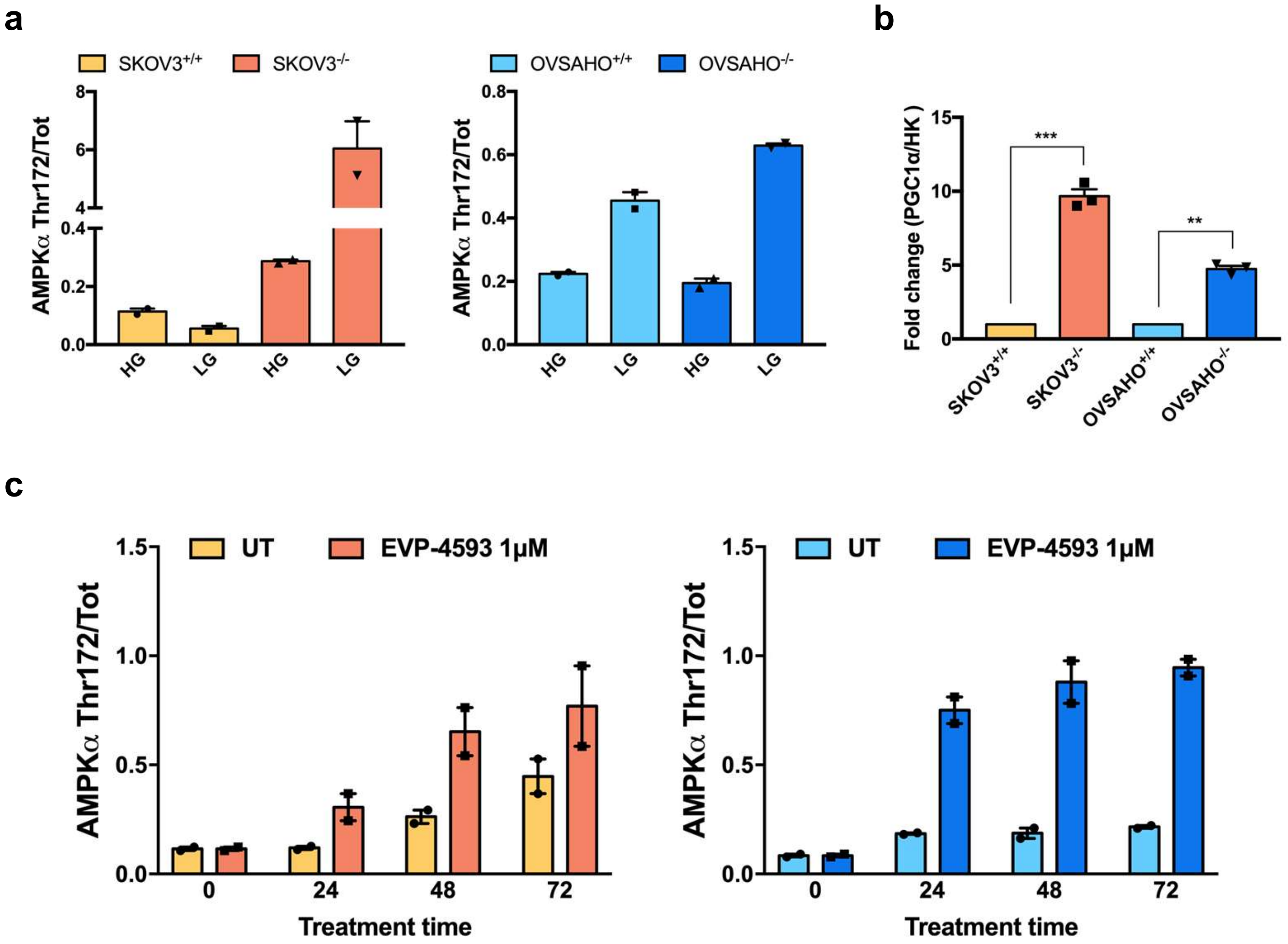

AMPK and PGC1α levels in CI impaired ovarian cancer models upon glucose restriction. (a) Densitometric analysis of phosphorylated (T172) and total AMPKα levels on lysates from SKOV3$^{+/+}$ (n=2), SKOV3$^{-/-}$ (n=2), OVSAHO$^{+/+}$ (n=2) and OVSAHO$^{-/-}$ (n=2) cells under 25mM (HG) and 5mM (LG) glucose growth conditions. HSP70 is used as loading control. Data (mean±SEM) are expressed as fold of AMPKα T172 phosphorylation to total AMPKα. (**b**) qRT-PCR analysis of total PGC1α gene expression in NDUFS3$^{-/-}$ compared to NDUFS3$^{+/+}$ OC cells in LG glucose growth condition (n=3). For each cell line, the data are expressed as fold change of the CI-competent cells and represented as mean±SEM. HK (housekeeping gene) **(c)** Densitometric analysis of phosphorylated (T172) and total AMPKα levels on lysates from SKOV3 (n=2) and OVSAHO cells (n=2) in LG glucose growth condition and 1μM EVP-4593. HSP70 is used as loading control. Data (mean±SEM) are expressed as fold of AMPKα T172 phosphorylation to total AMPKα.

Supplementary Fig. 2

The original figures and the full-length blots of Fig. 2b and c, respectively.

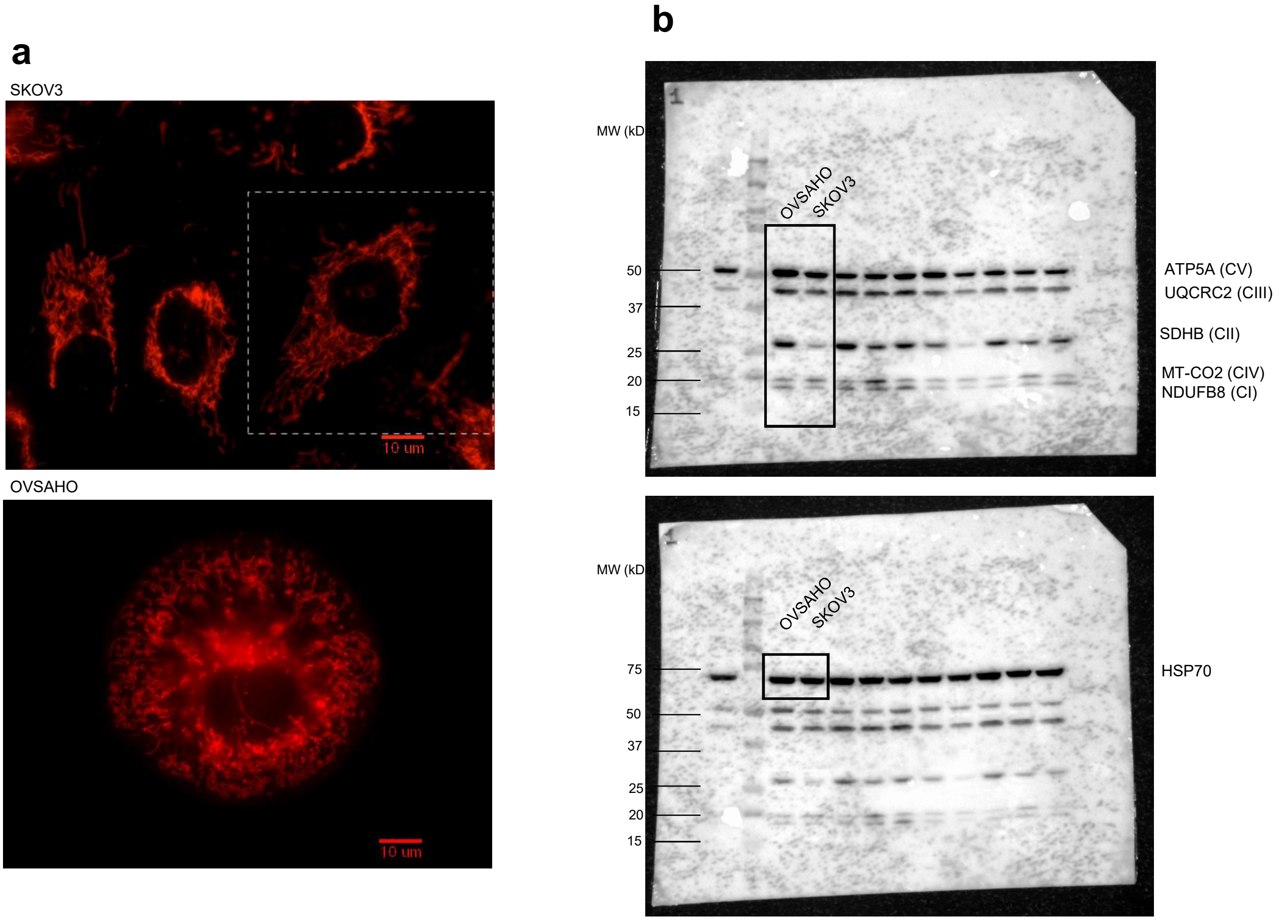

Supplementary Fig. 3

The full-length blots Fig. 3a and the full-length gels of Fig. 3b.

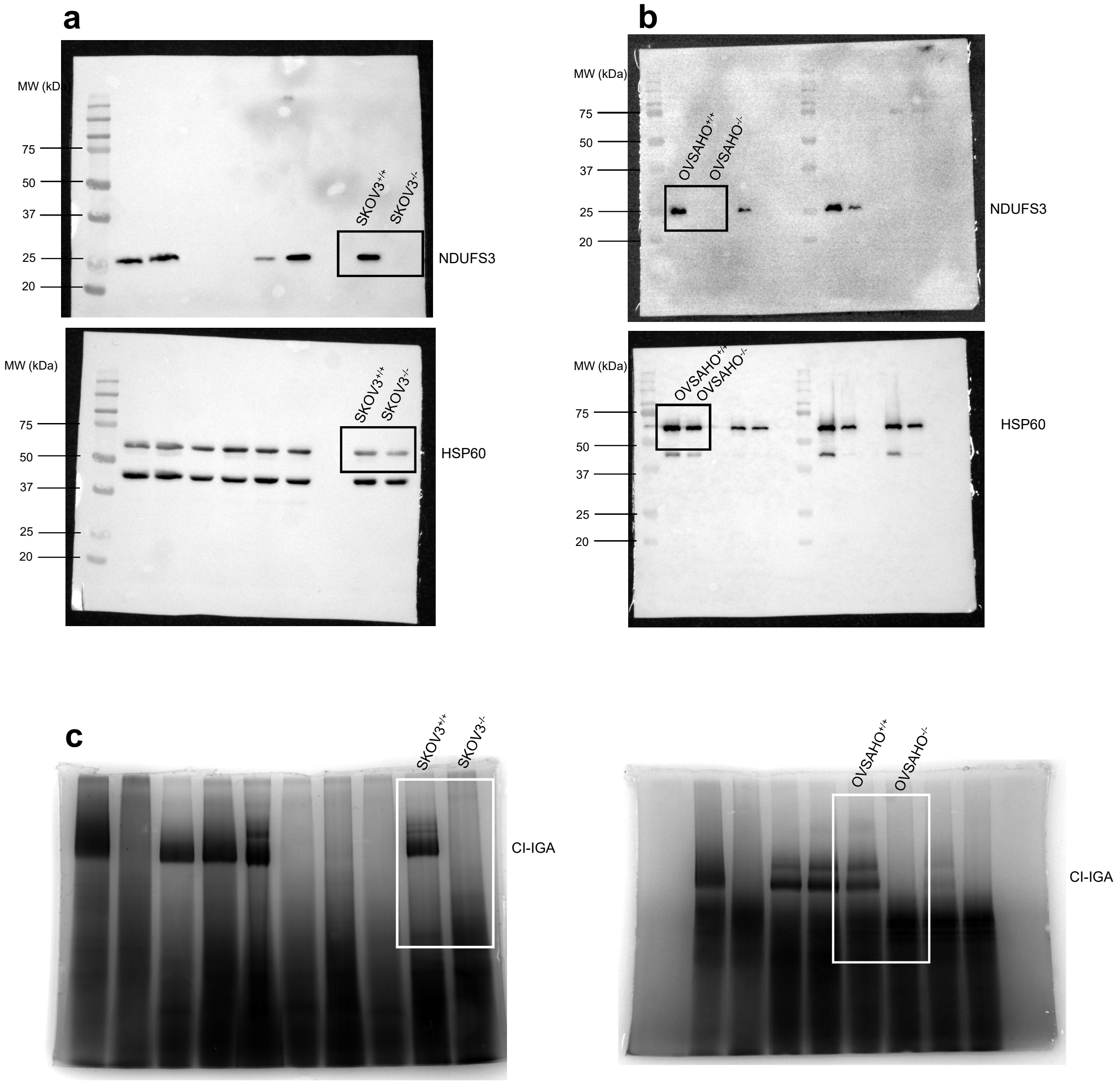

Supplementary Fig. 4

The full-length blots of Fig. 3f.

a. SKOV3

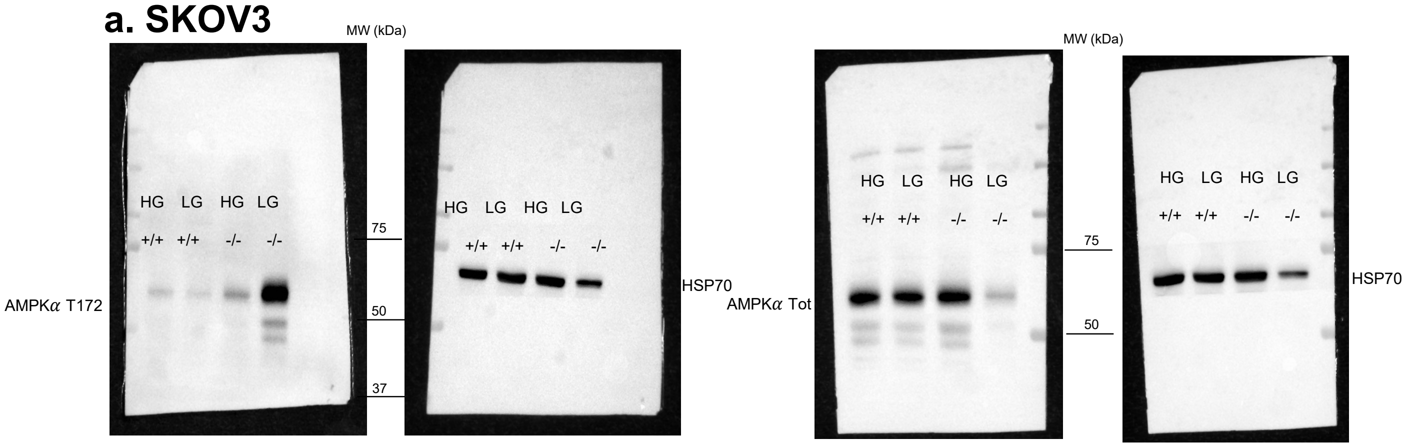

b. OVSAHO

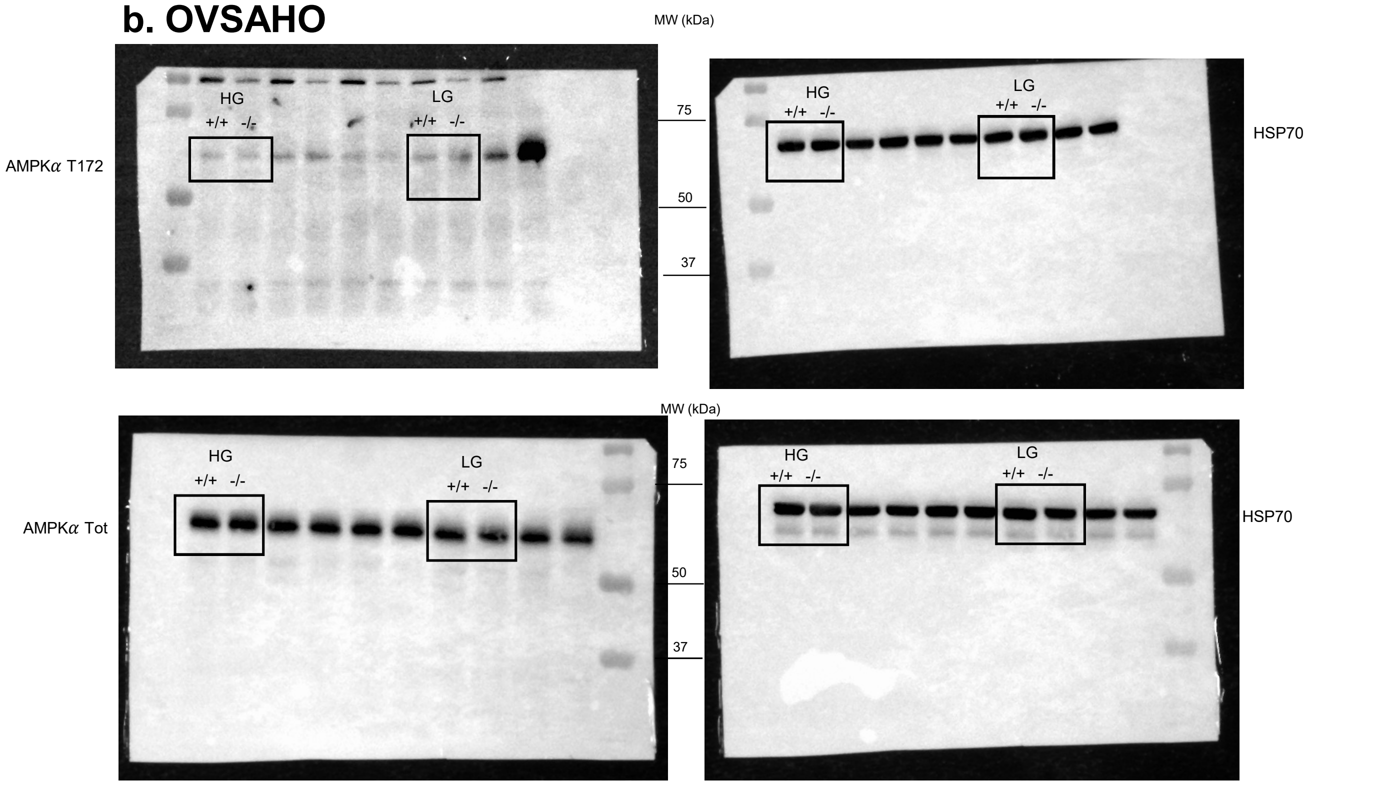

Supplementary Fig. 5

The full-length blots of Fig. 4b.

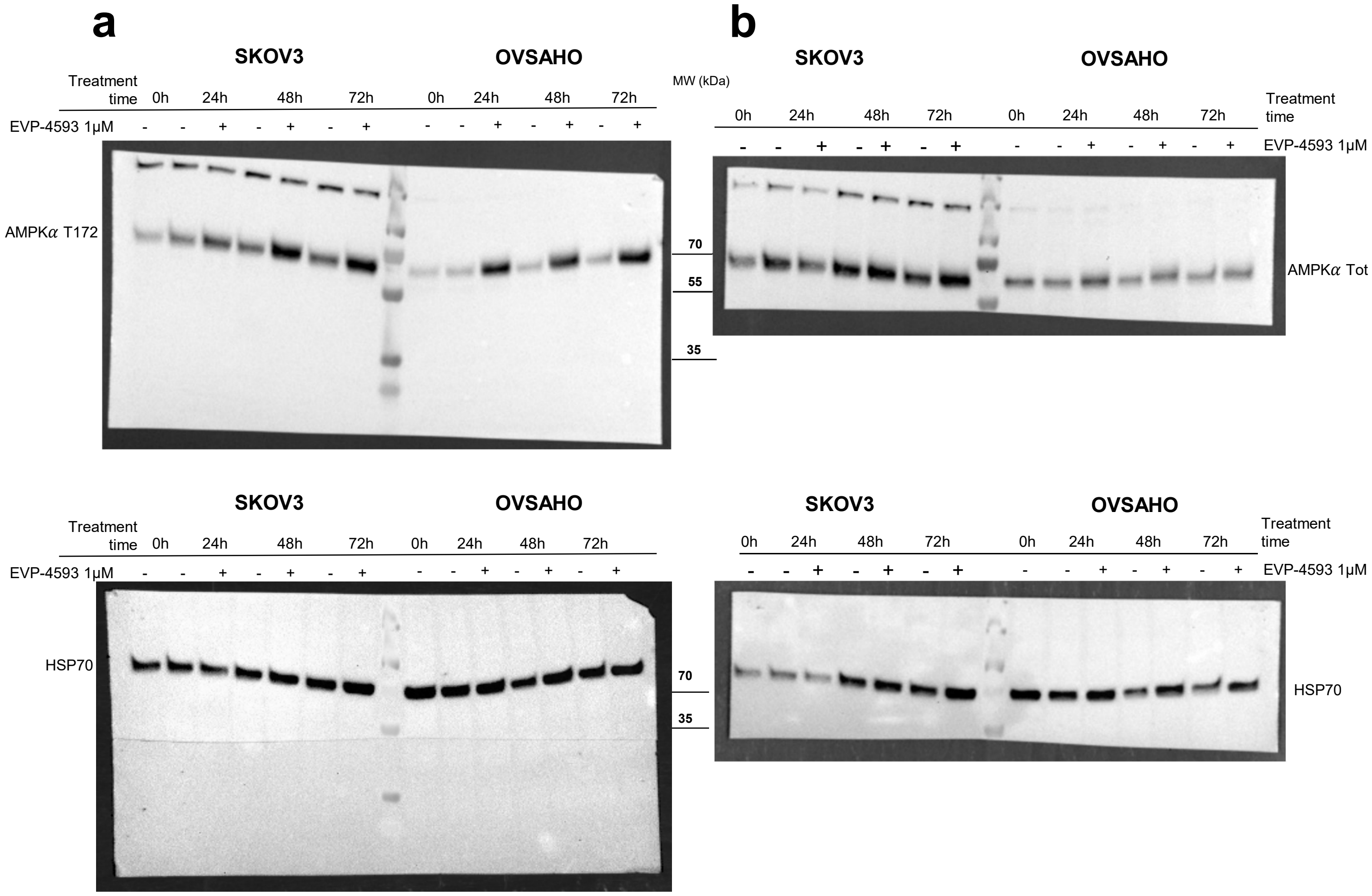

Supplementary Table 1. Gene name and primer sequences used for qRT-PCR with SYBR Green assay

Gene Name	Primer sequences	Conc. in PCR (μM)
COX5B	F: 5'-ACTGGGTTGGAGAGGGAGAT-3'	0.4
	R: 5'-TGGAGATGGAGGGGACTAAA-3'	0.4
PGC1α	F: 5'-GCCAAACCAACAACTTTATCTCTTC-3'	0.4
	R: 5'-CACACTTAAGGTGCGTTCATAAGTC-3'	0.4
TUBG1	F: 5'-TGCTGTGTCACTCCATTGCT-3'	0.4
	R: 5'-GTAAGGCTGGACCACCACAT-3'	0.4
GUSB	F: 5'-GAAAATACGTGGTTGGAGAGCTCATT-3'	0.4
	R: 5'-CCGAGTGAAGATCCCCTTTTTA-3'	0.4

Supplementary Table 2. Gene name, primer and probe sequences used for qRT-PCR with TaqMan assay

Gene Name	Primer and probe sequences	Conc. in PCR (μM)
ACADM	Primer 1: 5′-AGGCTCTGATGTAGCTGGTAT-3′<br>Primer 2: 5′-AGGAGCTTTAGGATCTGGATCA-3′<br>Probe: 5'-/56-FAM/AGGAGATGA/ZEN/GTATATTATTAATGGTCAGAAGATGTGGA/3IABkFQ/-3'	0.4
ACTB	Primer 1: 5'-ACAGAGCCTCGCCTTTG-3'<br>Primer 2: 5'-CCTTGCACATGCCGGAG-3'<br>Probe: 5'-/5Cy5/TCATCCATGGTGAGCTGGCGG/3IAbRQSp/-3'	0.4
ESRRA	Primer 1: 5'-CTATGGTGTGGCATCCTGTG-3'<br>Primer 2: 5′-AGGAGCTTTAGGATCTGGATCA-3′<br>Probe: 5'-3'	0.4
U2AF2	Primer 1: 5'-GTGCAGATTAACCAGGACAAGA-3'<br>Primer 2: 5′-CCTGCGGATCTTTAGTGACTG-3′<br>Probe: 5'-/5Cy5/TGGACGAGACTACCCAGGCTATGG/3IAbRQSp/-3'	0.4
